# The role of vitamin D in the management and rehabilitation of gestational diabetes mellitus

**DOI:** 10.3389/fnut.2026.1817924

**Published:** 2026-05-13

**Authors:** Ting Zhang, Qian Zhou, Lulu Shen, Song Gao

**Affiliations:** 1 College of Physical Education and Health Sciences, Zhejiang Normal University, Jinhua, China; 2 University Hospital, Zhejiang Normal University, Jinhua, China; 3 Physical Education Teaching Group, The First Experimental School of Shijiazhuang High-tech Industrial Development Zone, Shijiazhuang, China; 4 Jinhua Deren Rehabilitation Equipment Corporation, Jinhua, China

**Keywords:** gestational diabetes, insulin resistance, prevention, treatment, vitamin D

## 1 Introduction

Gestational diabetes mellitus (GDM) is characterized by abnormal glucose tolerance during pregnancy and is considered to be one of the most common pregnancy complications (1). It is evident that GDM has a detrimental effect on the short-term and long-term health of both mother and baby. The short-term consequences of pregnancy include an elevated incidence of cesarean section, preeclampsia and macrosomia (2, 3). The long-term consequences of pregnancy include an augmented risk of gestational diabetes, obesity in offspring and impaired glucose tolerance (4). The risk factors associated with GDM include advanced age, obesity, and a family history of type 2 diabetes (5, 6). Research has indicated a correlation between vitamin D levels during pregnancy and glucose metabolism, thereby suggesting a potential involvement of vitamin D in the development of GDM (7, 8). It is estimated that 1 billion people worldwide suffer from vitamin D deficiency, a condition that is particularly prevalent among pregnant women (9). A study of global maternal and neonatal vitamin D levels was conducted, the results of which indicated that the proportion of vitamin D deficiency (25(OH)D < 50 nmol/L) during pregnancy was found to be as follows: 87% in Southeast Asia, 83% in the Western Pacific, 64% in America, 57% in Europe and 46% in the Eastern Mediterranean (10).

There is a divergence of opinion regarding the correlation between vitamin D levels and GDM during pregnancy. A nested case-control study (200 pregnant women with GDM and 200 pregnant women with normal glucose tolerance) found that the incidence of vitamin D deficiency in pregnant women with GDM was significantly higher than that in pregnant women with normal glucose tolerance during the 26th to 28th week of pregnancy (11). The risk of GDM in subjects with 25(OH)D levels of less than 25 nmol/L is 1.8 times higher than that in subjects with higher vitamin D levels (11). A prospective observational study of 392 pregnant women in India found that the incidence of GDM in the 25th percentile ( ≤ 23.6nmol/L) was twice as high as that in the other quartiles (12). The risk of GDM in pregnant women with vitamin D deficiency during the 10–14 weeks of pregnancy was found to be 2.82 times higher (13). The risk of GDM in women with persistent vitamin D deficiency during the first trimester (10–14 weeks) and the second trimester (15–26 weeks) of pregnancy increased by 4.46 times (13).

However, other studies have demonstrated an absence of a significant correlation between the two variables (14). A cohort study based on multi-ethnic groups was conducted to observe the relationship between 25(OH)D and GDM in 745 multi-ethnic pregnant women during 15–28 weeks of pregnancy. Following the adjustment of age, parity, education level and seasonal factors, serum 25(OH)D levels of less than 50 nmol/L demonstrated a correlation with GDM (OR = 1.6, 95% CI = 1.1–2.2). However, this correlation disappeared following additional adjustment for variables reflecting fat mass (skinfold thickness or body mass index) and race (15). An Indian study determined glucose metabolism indexes such as serum 25(OH)D, glucose tolerance and plasma insulin at 30 weeks of pregnancy, and found that vitamin D had no association with GDM (16). Park et al. (17) measured serum 25-OH-D in pregnant women at 12–14, 20–22 and 32–34 weeks of gestation, respectively, and screened for GDM at 24–28 weeks of pregnancy. The findings indicated that the risk of GDM, insulin resistance and β cell dysfunction was not associated with serum 25(OH)D levels. A prospective cohort study of 785 pregnant women conducted by Australian scholars also failed to demonstrate a relationship between vitamin D deficiency or deficiency and GDM development (14). The observed variations in the aforementioned research results are attributable to a multitude of factors, including but not limited to sample size, race, region, season, age, and living habits across diverse studies.

Vitamin D deficiency is prevalent among pregnant women. The role of low vitamin D status in GDM has attracted increased attention in recent years. It has been demonstrated that vitamin D has the capacity to enhance insulin sensitivity and augment the functionality of islet B cells (18). The global prevalence of low vitamin D status, in conjunction with the escalating incidence of GDM, when evaluated from the perspective of health economics, suggests a potential benefit from the supplementation of appropriate vitamin D during pregnancy or prior to conception. This paper posits that vitamin D plays a pivotal role in the treatment and prevention of GDM.

## 2 Criteria for determining vitamin D deficiency

Vitamin D is a fat-soluble vitamin that exists in two forms: vitamin D2 and vitamin D3. Research has shown that vitamin D3 accounts for 90%−95% of the total vitamin D in the human body (19). The primary source of vitamin D2 is dietary intake, whereas vitamin D3 is predominantly synthesized in the skin through ultraviolet irradiation. This process is considered the predominant pathway through which the human body acquires vitamin D. Subsequent to this, vitamin D undergoes conversion to 25(OH)D through secondary hydroxylation in the liver and kidneys within the organism. This process is pivotal in the execution of its biological function. Furthermore, vitamin D is classified as a prohormone, which can influence calcium and phosphorus metabolism, immune regulation, and anti-inflammation (20, 21).

Currently, there is an absence of criteria for evaluating vitamin D status. According to the Institute of Medicine (IOM) of the National Academy of Sciences, Serum 25(OH)D concentrations of less than 30 nmol/L (12 μg/L) are indicative of deficiency; concentrations ranging from 30 to 50 nmol/L (12–20 μg/L) are considered insufficient; concentrations of more than 50 nmol/L (20 μg/L) are considered sufficient; and concentrations exceeding 125 nmol/L (50 μg/L) are considered to pose a potential risk of poisoning (22). The American Endocrine Association has defined serum 25(OH)D levels of less than 50 nmol/L (20 μg/L) as indicative of deficiency, based on the musculoskeletal function of vitamin D. 52.5–72.5 nmol/L (21–29 μg/L) is considered insufficient, while ≥75 nmol/L (30 μg/L) is considered sufficient (23). The National Osteoporosis Society (NOS) has established the following serum 25(OH)D thresholds with the focus on bone health: 30 nmol/L for deficiency; 30–50 nmol/L for insufficient; and >50 nmol/L for sufficient (24).

## 3 Mechanism of vitamin D regulating glucose homeostasis

It is an established fact that a series of physiological changes occur during a healthy maternal pregnancy. In order to maintain the balance of glucose, the secretion of insulin is stimulated by hypertrophy and proliferation of islet β cells, as well as increasing glucose (25). However, during GDM, the body experiences a chronic resistance to insulin, resulting in insufficient secretion of the pancreas in response to peripheral insulin resistance. This, in turn, causes dysfunction of β cells and fails to meet the metabolic needs of pregnancy, thus leading to hyperglycaemia (26, 27). The pathological mechanisms of GDM and type 2 diabetes share numerous similarities (28). Current data suggest a correlation between vitamin D deficiency and insulin resistance, as well as β cell dysfunction. Supplementation with vitamin D has been demonstrated to enhance glucose clearance rate and insulin secretion in the body (29).

Vitamin D has been demonstrated to impact glucose homeostasis through multiple mechanisms: (1) the alterations in pancreatic function observed in diabetic patients may be attributable to inflammation of islet cells and the infiltration of immune cells (30). It has been demonstrated that vitamin D possesses anti-inflammatory properties and has the capacity to promote the recovery of insulin secretion (31); (2) the process of receptor-mediated endocytosis, driven by the insulin receptor, is subject to regulation by intracellular calmodulin (32). It has been demonstrated that vitamin D enhances the absorption of calcium in the duodenum and kidneys, thereby facilitating the activation of intracellular signal transduction by insulin (33); (3) the presence of vitamin D receptors on the surface of pancreatic β cells has been demonstrated. It has been hypothesized that vitamin D may regulate insulin secretion by pancreatic β cells (34).

## 4 Therapeutic effect of vitamin D on GDM

Despite the controversy surrounding the correlation between vitamin D and GDM, vitamin D is regarded as a potential pharmaceutical agent for the treatment of GDM (35, 36). A study by Asemi et al. (35) randomly divided 54 pregnant women with GDM into two groups, receiving a vitamin D supplement or placebo, respectively. The pregnant women who received the vitamin D supplement (*n* = 27) were administered 50,000 U of vitamin D on the day of enrolment (day 0) and on day 21. The placebo group (*n* = 27) was also administered a placebo. During this period, all participants maintained their usual levels of daily physical activity and dietary intake, and did not take other nutritional supplements. Following a period of 6 weeks, a significant decrease in the fasting blood glucose, serum insulin and insulin resistance of pregnant women in the intervention group was observed. The administration of vitamin D supplements to pregnant women with GDM has been demonstrated to reduce the incidence of polyhydramnios and neonatal hyperbilirubinemia. However, a contradictory study found that vitamin D supplementation can reduce fasting blood glucose, but did not find a significant improvement in insulin and insulin resistance (36). Yin et al. (37) conducted a meta-analysis investigating the impact of vitamin D supplementation on GDM. Their results showed that vitamin D supplementation can prevent and treat insulin resistance and beta cell dysfunction by reducing fasting blood glucose and insulin levels and increasing insulin sensitivity.

Of course, there are also different research results. Mirzaei-Azandaryani et al. (38) conducted a randomized controlled trial with three blinding methods. They randomly divided 88 pregnant women with vitamin D levels below 30 ng/mL into a vitamin D group and a control group at 8–10 weeks of pregnancy. There were 44 women in each group. The vitamin D group took 4,000 IU of vitamin D tablets daily for 18 weeks, while the control group took placebo tablets. The results showed that vitamin D supplementation did not improve fasting blood glucose or insulin levels, insulin resistance, or the incidence of gestational diabetes. As the current studies have small sample sizes and inconsistent results, more randomized controlled studies are needed to clarify the optimal dosage and treatment time for preventing GDM.

In addition to vitamin D alone, the combination of vitamin D with other nutrients has emerged as a promising therapeutic approach for GDM. A research team in Iran randomly divided 60 GDM pregnant women not receiving insulin treatment into two groups (39). The control group was administered a placebo, while the experimental group was given magnesium-zinc-calcium-vitamin D combined supplement for a period of 6 weeks. During the course of the study, all participants were required to maintain their usual dietary habits and levels of physical exercise. The findings demonstrated that a combination of magnesium, zinc, calcium, and vitamin D supplementation led to a substantial reduction in fasting blood glucose levels, insulin resistance, and serum high-sensitivity C-reactive protein concentrations. Furthermore, the experimental group exhibited a reduced incidence of macrosomia (3.3 vs. 16.7%, *P* = 0.08) and lower neonatal weight [(3,089.8 ± 519.9)g vs. (3,346.3 ± 411.1) g, *P* = 0.05]. A further randomized controlled study involved 56 pregnant women with GDM who did not require insulin treatment. The experimental group was administered 100 mg of calcium daily, along with 50,000 U of vitamin D at baseline and on day 21. In contrast, the placebo group received a placebo at the aforementioned time points. The findings indicated a substantial decline in the fasting blood glucose, serum insulin levels, and insulin resistance levels of pregnant women within the experimental group (40). A study by Karamali et al. (41) found that compared with GDM pregnant women in placebo group, the cesarean section rate and hospitalization rate of pregnant women supplemented with calcium and vitamin D were significantly lower. There was no macrosomia in the experimental group, and the incidence of macrosomia in the placebo group was 13.3%. This finding indicated that the combination of vitamin D with other nutrients may have a positive effect on GDM pregnant women.

## 5 Prevention of GDM by vitamin D

The present study hypothesizes that vitamin D supplementation may reduce the risk of GDM (42, 43). A number of studies have indicated that the positive rates of the glucose provocation test (34.8 vs. 11.4%) and glucose tolerance test (35.6 vs. 10.9%) in pregnant women who did not take vitamin D during the early and middle stages of pregnancy were significantly higher than those of pregnant women who took 5,000 IU of vitamin D daily (44, 45). The physiological changes associated with GDM manifest during the first trimester, suggesting that vitamin D supplementation in pregnant women during this early stage may serve as a preventative measure against GDM (6, 13, 46).

A subsequent study conducted within the last decade has indicated that, in comparison with pregnant women who did not ingest vitamin D supplements, those who consumed 1–399 IU or more than 400 IU per day of vitamin D supplements exhibited a substantially diminished risk of GDM (RR values: 0.86 and 0.71) (47). Shahgheibi et al. (44) conducted a randomized controlled trial on 100 pregnant women with at least one GDM risk factor in the first trimester. The pregnant women in the intervention group took 5,000 IU vitamin D every day until the 26th week of pregnancy, while the control group took a placebo. The results showed that the incidence of GDM in pregnant women in the intervention group was significantly lower than that in the control group (11.4% compared with 34.8; *P* = 0.01). However, some RCT studies have not found the correlation between vitamin D supplementation and the risk of GDM (48). One study randomly divided 179 pregnant women with gestational age less than 20 weeks and serum vitamin D level less than 30 g/L into two groups (49). The intervention group was supplemented with 50,000 IU vitamin D every 2 weeks, while the control group was supplemented with 400U vitamin D every day until delivery. The results showed that the incidence of GDM in the two groups was 8 and 13% respectively (*P* = 0.25), which suggested that high-dose vitamin D supplementation from the 14th week of pregnancy could not improve the blood sugar level during pregnancy. Other small sample randomized controlled trials have not found that vitamin D intervention has a preventive effect on GDM (48, 50). Therefore, the number of preventive RCT studies on the risk of GDM caused by vitamin D supplementation is small, and more high-quality experimental studies on vitamin D and GDM can be carried out in the future.

## 6 Conclusion

Vitamin D deficiency is prevalent among pregnant women, with the level of vitamin D in pregnant women being influenced by numerous factors. The present research results are inconsistent with regard to the hypothesis that elevated levels of vitamin D during pregnancy may reduce the risk of GDM. The effective prevention and treatment of GDM has become a significant issue in the field of maternal and child nutrition on a global scale.

The present status of research regarding the role of vitamin D in the treatment and prevention of GDM remains controversial. The physiological changes associated with GDM manifest during the early stages of pregnancy (6, 13, 46); however, it is possible that by the time these changes are detected, the opportunity for intervention may have elapsed, rendering pregnancy or even the pre-pregnancy period the most opportune time for intervention. In previous studies, there has been a lack of uniform criteria for judging vitamin D, and the supplementary dose, mode and intervention cycle of vitamin D intervention trials are different, so it is difficult to evaluate the influence of intervention measures and many mixed factors on GDM. A substantial body of clinical research supports the notion that women diagnosed with GDM are more prone to vitamin D deficiency. However, the direct involvement of vitamin D in the pathogenesis of GDM, and the precise mechanism by which it does so, remain to be elucidated. Whether serum vitamin D levels in pregnant women can be used as a marker for identifying those at high risk of GDM still requires a large-scale prospective study. Therefore, future research must increase sample diversity, reliability and randomness, improve clinical persuasiveness and provide new ideas for predicting and preventing GDM. In the future, the design of high-quality multicentre randomized controlled experiments is necessary to explore the relationship between vitamin D and GDM. The preventive and therapeutic effects of vitamin D on GDM are worthy of investigation.
